# Transparent anti-fogging and self-cleaning TiO_2_/SiO_2_ thin films on polymer substrates using atmospheric plasma

**DOI:** 10.1038/s41598-018-27526-7

**Published:** 2018-06-25

**Authors:** Jean-Baptiste Chemin, Simon Bulou, Kamal Baba, Charly Fontaine, Thierry Sindzingre, Nicolas D. Boscher, Patrick Choquet

**Affiliations:** 1 Materials Research and Technology Department, Luxembourg Institute of Science and Technology, 5 Avenue des Hauts-Fourneaux, L-4362 Esch-sur-Alzette, Luxembourg; 2 AcXys Technologies, 50 bis Rue des Vingt Toises, 38950 Saint Martin le Vinoux, France

## Abstract

Transparent anti-fogging and self-cleaning coatings are of great interest for many applications, including solar panels, windshields and displays or lenses to be used in humid environments. In this paper, we report on the simultaneous synthesis, at atmospheric pressure, of anatase TiO_2_ nanoparticles and low-temperature, high-rate deposition of anatase TiO_2_/SiO_2_ nanocomposite coatings. These coatings exhibit durable super-hydrophilic and photocatalytic properties. The strategy followed relies on concomitant and separated injections of titania, i.e. titanium isopropoxide, and silica, i.e. hexamethyldisiloxane, precursors in the stream of a blown-arc discharge to form transparent anti-fogging and self-cleaning anatase TiO_2_/SiO_2_ nanocomposite coatings on polymer substrates.

## Introduction

Anti-fogging coatings are very attractive for many applications, such as windshields, where they can keep visibility high and improve driving safety, organic ophthalmic lenses and solar cells, where they can prevent performance loss^1^. Among the existing solutions^2,3^, numerous anti-fogging treatments or anti-fogging coatings have been proposed. These actions enhance the attractive forces between the water and surface in order to overcome surface tension, as well as dispersing the water droplets to form an invisible water layer. Nevertheless, the current solutions have many drawbacks. On one hand, anti-fogging sprays do not withstand washing and must be reapplied regularly. On the other hand, although permanent coatings have been developed to increase the surface energy and wettability, this high surface energy also makes them prone to surface contamination. Further, their super-hydrophilic/hydrophilic property disappears after several weeks or months. Therefore, the addition of a self-cleaning property can significantly enhance the potential of permanent coatings^4,5^, as well as reduce the costs related to environmental contamination. Several preparatory steps towards the creation of transparent anti-fogging and self-cleaning surfaces have already been investigated. Notably, the nanotexturing of silica via etching, broadly used in screens and lenses, can ensure the formation of such multifunctional surfaces^6^. Nevertheless, such an approach is strongly substrate-dependent and cannot easily be industrially implemented on large areas.

Among the permanent coatings studied, anatase titanium dioxide (TiO_2_)/silicon dioxide (SiO_2_) nanocomposite coatings can efficiently combine the super-hydrophilic property of SiO_2_ and the photocatalytic property of anatase TiO_2_^7,8^. If many deposition methods can contribute to the formation of each material, i.e. SiO_2_ and anatase TiO_2_, the formation of anatase TiO_2_/SiO_2_ nanocomposite coatings has mainly been achieved via wet chemistry routes and multistep procedures^7–9^. In addition, the deposition of anatase TiO_2_/SiO_2_ nanocomposite coatings is often based on the embedment of preformed anatase TiO_2_ nanoparticles in the SiO_2_ matrix, which can be grown by sol-gel coating or chemical vapor deposition. Dembele *et al*. have notably reported the incorporation of the preformed anatase TiO_2_ into a siloxane coating grown from the plasma-enhanced chemical vapor deposition (PECVD) of tetramethylorthosilicate (TMOS)^10^. Nevertheless, the manipulation of nanoparticles is a major drawback of this strategy.

PECVD, including atmospheric-pressure PECVD (AP-PECVD), which can readily lead to the deposition of dense SiO_2_ layers at room temperature^11^, can also contribute to the formation of anatase TiO_2_ in both powder^12^ and thin-film forms^13^. Interestingly, AP-PECVD processes can significantly lower the temperature usually required to form anatase TiO_2_ via CVD^14^, and ensure its compatibility with polymer substrates^15,16^. In this work, we explore the simultaneous synthesis and high deposition rate of anatase TiO_2_/SiO_2_ nanocomposite thin films on polymer foils using an atmospheric pressure blown-arc discharge with two affordable titanium and silicon precursors. Different precursor ratios were investigated in order to tune the chemical composition and physical properties of the anatase TiO_2_/SiO_2_ nanocomposite coatings. The functional properties of the coatings, i.e. super-hydrophilicity/anti-fogging, photo catalysis/self-cleaning and optical transparency, and their durability were assessed.

## Results and Discussion

### One-pot synthesis and deposition of anatase TiO_2_/SiO_2_ nanocomposite coatings

**Figure 1:**
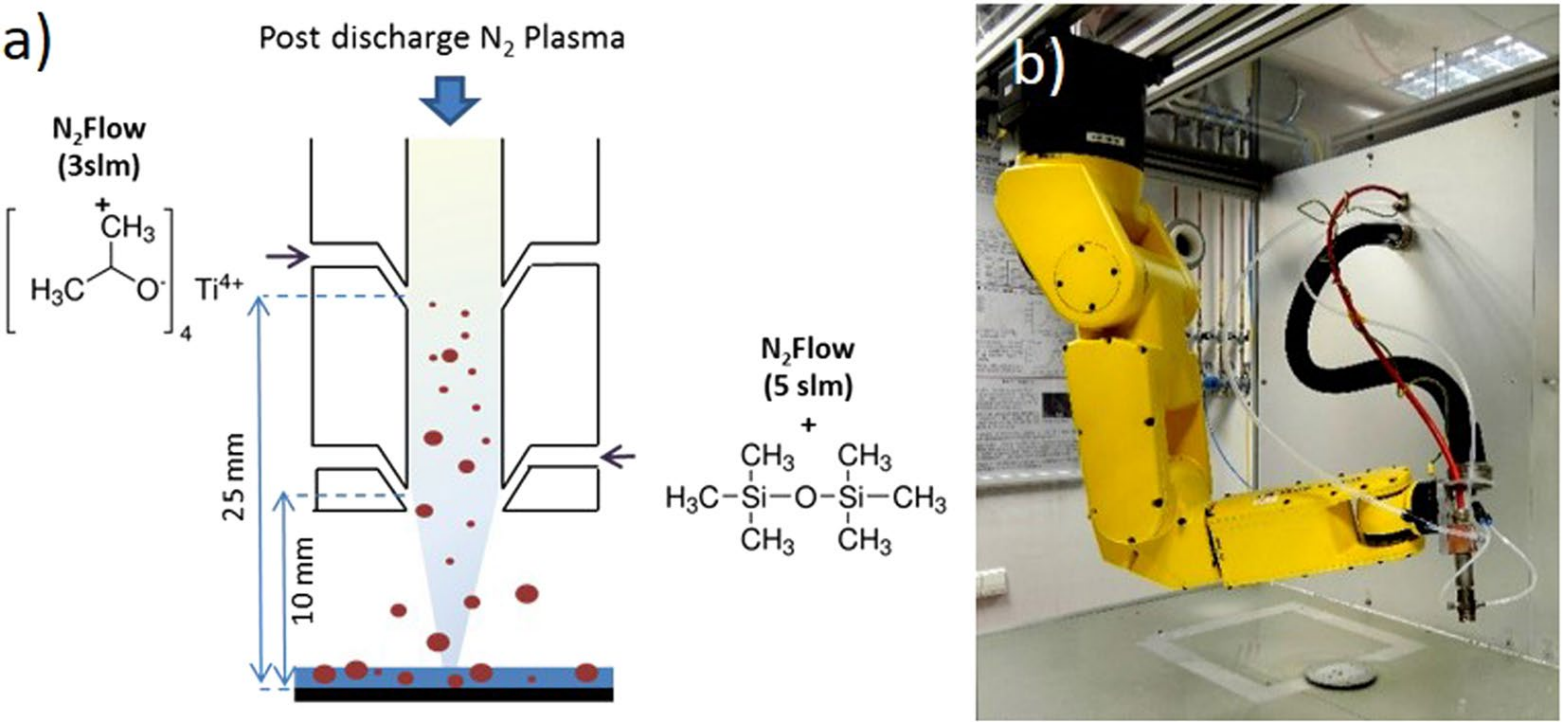
(**a**) Schematic showing the separate injection of the TiO_2_ and SiO_2_ precursors into the stream of a blown arc discharge, (**b**) photography of the experimental set-up presenting the torch mounted on a 6-axis arm robot.

Our strategy towards the simultaneous synthesis of anatase TiO_2_ nanoparticles and their embedment into a SiO_2_ matrix deposited at low substrate temperature relies on the separate injection of the TiO_2_ and SiO_2_ precursors into the stream of a blown arc discharge (Fig. 1a). Due to its rather high reactivity and its subsequent ability to form anatase TiO_2_ nanoparticles in AP-PECVD processes^12^, titanium (IV) isopropoxide (TTIP) was chosen as the TiO_2_ precursor. As depicted in Fig. 1a, TTIP was injected at the nearest position to the outlet of the blown plasma discharge nozzle. In this part of the post-discharge, gas phase reactions and the subsequent formation of crystalline nanoparticles are promoted by the high density of excited species and the relatively high gas temperature. In order to optimize the production of anatase TiO_2_ nanoparticles, the TTIP flow was maintained at 6 µL·min^−1^, which in the present condition was the highest possible flow without troublesome condensation of the TTIP precursor in the nebulization system. On the other hand, hexamethydisiloxane (HMDSO) was selected as the SiO_2_ thin film precursor. HMDSO, which has been widely investigated in AP-PECVD processes^17^, was injected into the lower part of the stream of the blown-arc discharge close to the substrate in order to promote heterogeneous chemical reactions at the surface of the substrate with the perspective of growing an SiO_2_ layer that would possibly embed the crystalline TiO_2_ nanoparticles formed above the first injection. In the present study, the HMDSO flow was varied from 0 to 10 µL·min^−1^ in order to investigate various thin film compositions.

**Figure 2:**
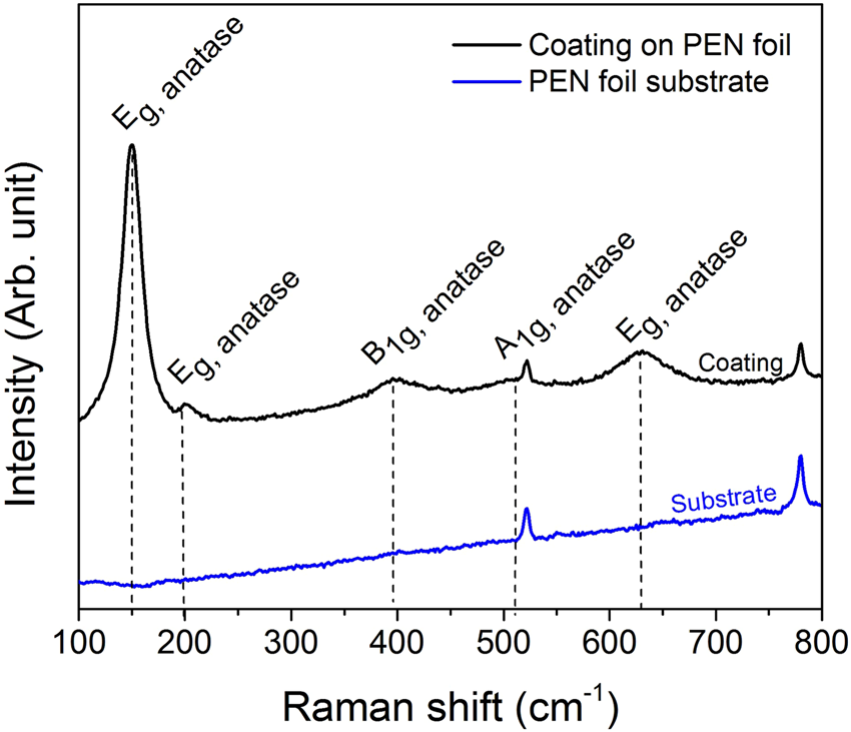
Raman spectrum of the anatase TiO_2_/SiO_2_ nanocomposite coating elaborated from a 2 µL·min^−1^ HMDSO delivery rate on a PEN substrate. The Raman spectrum of an uncoated PEN foil is also plotted as a reference.

The simultaneous reactions of TTIP and HMDSO with the investigated blown plasma discharge led to the deposition of homogeneous coatings across the entire surface of the substrate. No visually noticeable crack was observed on their surface. When deposited on poly(ethylene 2,6-naphthalate) (PEN) foils, the films appeared transparent and were indistinguishable from each other. When deposited on silicon wafers, the prepared coatings with closer thicknesses exhibited a different color for each conditions of the HMDSO delivery rates investigated. This color change, due to interference fringes, suggests thickness and/or refractive index variation between the coatings. Irrespective of the HMDSO delivery rate, most of these cluster were composed of anatase TiO_2_ such as highlighted by Raman spectroscopy analysis with the presence of bands at 144, 197, 399 and 640 cm^−1^ (Fig. 2). The band at 522 cm^−1^ and 780 cm^−1^ are assigned to the polymer PEN substrate. No rutile TiO_2_ or other phase were detected. The absence of the Raman shift of SiO_2_ is due to the low Raman signal of the amorphous SiO_2_ thin film^18^. Electron diffraction and high-resolution transmission electron microscopy obtained by Transmission Electron Microscopy (not shown here) highlighted the formation of anatase crystallites with a size close to 10 nm as already observed in one of our previous article^12^.

### Characterization of the anatase TiO_2_/SiO_2_ nanocomposite coating

**Figure 3:**
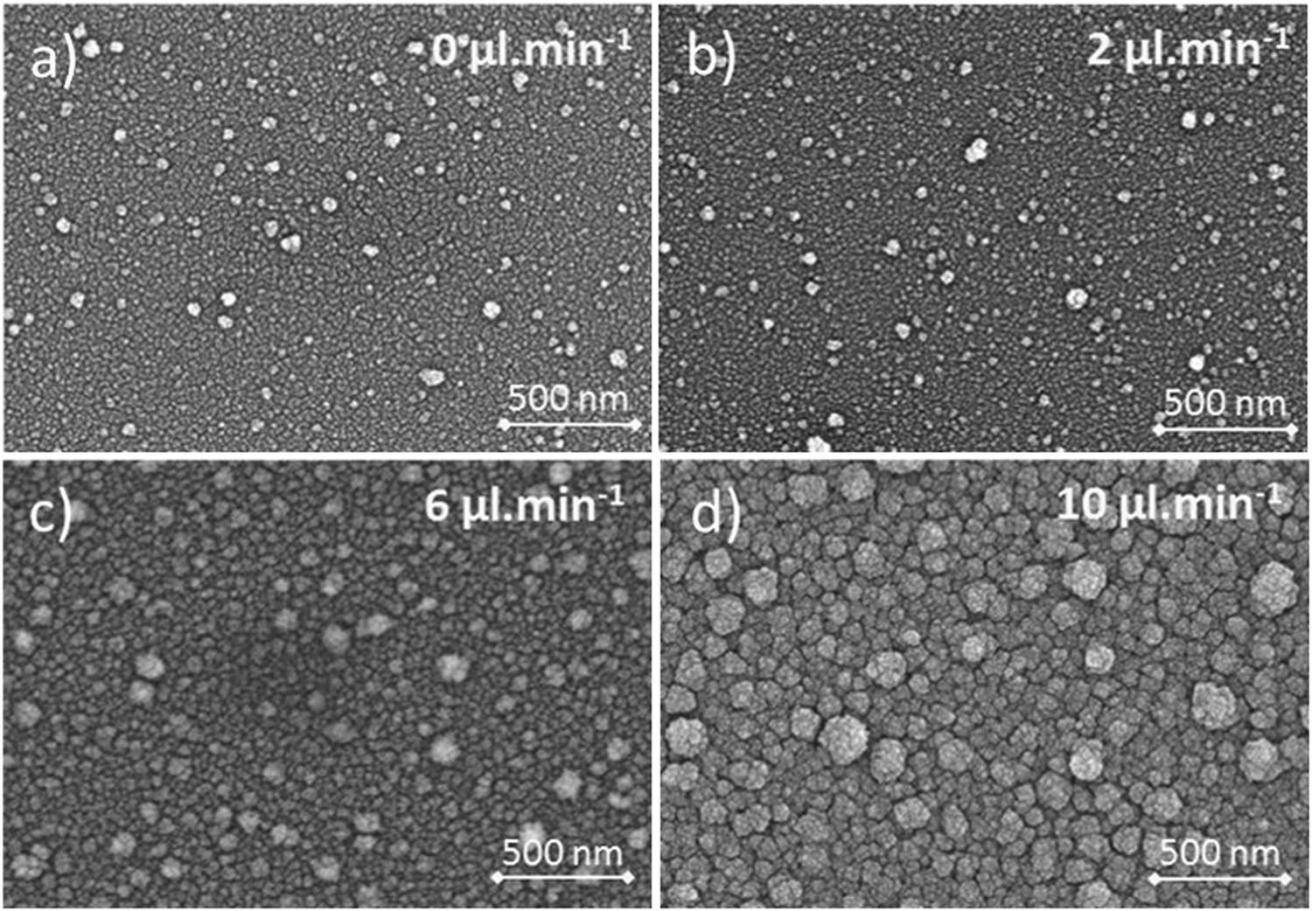
High-magnification (x50,000) scanning electron micrographs of the coatings elaborated from various HMDSO delivery rates: (**a**) 0 µl.min^−1^, (**b**) 2 µl.min^−1^, (**c**) 6 µl.min^−1^, (**d**) 10 µl.min^−1^.

Scanning electron microscopy (SEM) observations confirmed that the coatings were dense and homogeneous, and fully covered the substrate surface. Similar to a previous work focusing on the deposition of TiO_2_ coatings with the same setup^12^, SEM analyses of films deposited without HMDSO revealed a film structure composed of grains ranging in size from 10 to 50 nm. Up to a 2 µL·min^−1^ HMDSO delivery rate, the morphology of the deposited coatings was not significantly different to that grown without HMDSO (Fig. 3b). A further increase of the HMDSO delivery rate slowly led to a conversion of the microstructure of the films toward a cauliflower morphology (Fig. 3c,d).

**Figure 4:**
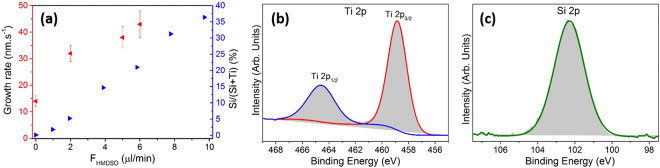
(**a**) Growth rates and Si/(Si + Ti) ratios for the films elaborated from the various HMDSO delivery rates investigated (F_HMDSO_). High-resolution spectra of the Ti 2p (**b**) and Si 2p core levels (**c**) for the XPS surface analysis of the anatase TiO_2_/SiO_2_ nanocomposite coating elaborated from a F_HMDSO_ = 10 µL·min^−1^.

Unsurprisingly, the growing addition of the HMDSO precursor to the deposition process led to an increase of the deposition rate of the films. The thickness of the films, evaluated by SEM side-view observation (Figure S1), ranged from 35 nm (F_HMDSO_ = 0 µL·min^−1^) to 110 nm (F_HMDSO_ = 6 µL·min^−1^), which corresponds to a growth rate variation of between 14 nm.s^−1^ and 43 nm.s^−1^(Fig. 4a).

X-ray photoelectron spectroscopy (XPS) showed that all the synthesized films contain titanium, silicon, and oxygen with the concentration depending on the HMDSO delivery rate. The nitrogen and carbon contents were both measured at below one atomic percent after the etching of the films. Reasonably and similarly to the measured growth rates, the silicon concentration of the films also increased while adding a more important flow of HMDSO into the discharge. The ratio of the silicon content over the total amount of silicon and titanium (Si/(Si + Ti)) increased linearly from 0 to 36% when increasing the HMDSO delivery rate from 0 to 10 µL·min^−1^, respectively (Fig. 4a).

The core level spectra of Ti2p (Fig. 4c) and Si2p (Fig. 4b) were investigated in order to unveil the chemical environment of the silicon and titanium elements.

Irrespective of the deposition conditions (i.e. HMDSO flow), the positions of the Ti 2p_1/2_ and Ti 2p_3/2_ peaks, observed at 464.5 eV and 458.8 eV (Fig. 4b), respectively, is characteristic of TiO_2_ bondings This value is very close to that of pure TiO_2_ generally reported^8,15,19,20^, indicating that Ti is predominantly involved in Ti-O-Ti bonding. Nevertheless, Ti-O-Si bonding can not be excluded as the peak shows a significant full width at half maximum (FWHM) of 1.5 eV.

High resolution spectra of Si 2p core level (Si 2p_1/2_ and Si 2p_3/2_) was measured at 102.3 eV (Fig. 4c). No evidences of titanium silicide phase, i.e. Si-Ti metallic bonds at 98.6 eV is observed. Si 2p peak appears relatively broad with a FWHM of 2.1 eV. The peak energy of the Si 2p, as well as the relatively large FWHM suggests the existence of O-Si-O and Si-O-Ti bonding, which is generally reported in the literature around 102.5 eV (Fig. 4c)^17^.

According to these results, the obtained coatings are much likely composed of pure TiO_2_ particles embedded in a SiO_2_ like matrix. The SiO_2_ like coating is much likely covering the TiO_2_ particles. It acts as a solid matrix, binding TiO_2_ particles into the coating, thus explaining the existence of both Si-O-Si and Si-O-Ti bonding.

Because TTIP is injected first and in the nearest position into the flowing glow discharge, homogeneous reactions are promoted and lead to the synthesis of anatase containing pure TiO_2_ particles. Then, the formed TiO_2_ particles goes through HMDSO injection. This favors heterogeneous reactions of HMDSO on TiO_2_ particles, and thus SiO_2_ like deposition on the formed titania nanoparticles. Besides, heterogeneous reactions of HMDSO and the substrate surface further reinforce the cohesion of the nanocomposite TiO_2_/SiO_2_ coating.

These conclusions contrast with results obtained during preliminary experiments of this work, which only allowed to form a single SiTiO_x_ phase^21^ to be formed when injecting the TTIP and HMDSO precursors in the same area of the plasma post-discharge (unpublished work). Thus, a separate injection of the TTIP and HMDSO precursors, such as that described above, is necessary to generate a two-phase structure with the crystalline TiO_2_ phase separated from the SiO_2_ phase. The prepared films can thus be described as anatase TiO_2_ cristallites embedded in a SiO_2_ like matrix, i.e. TiO_2_/SiO_2_ nanocomposite coatings.

### Super-hydrophilicity, photocatalytic activity and durability

**Figure 5:**
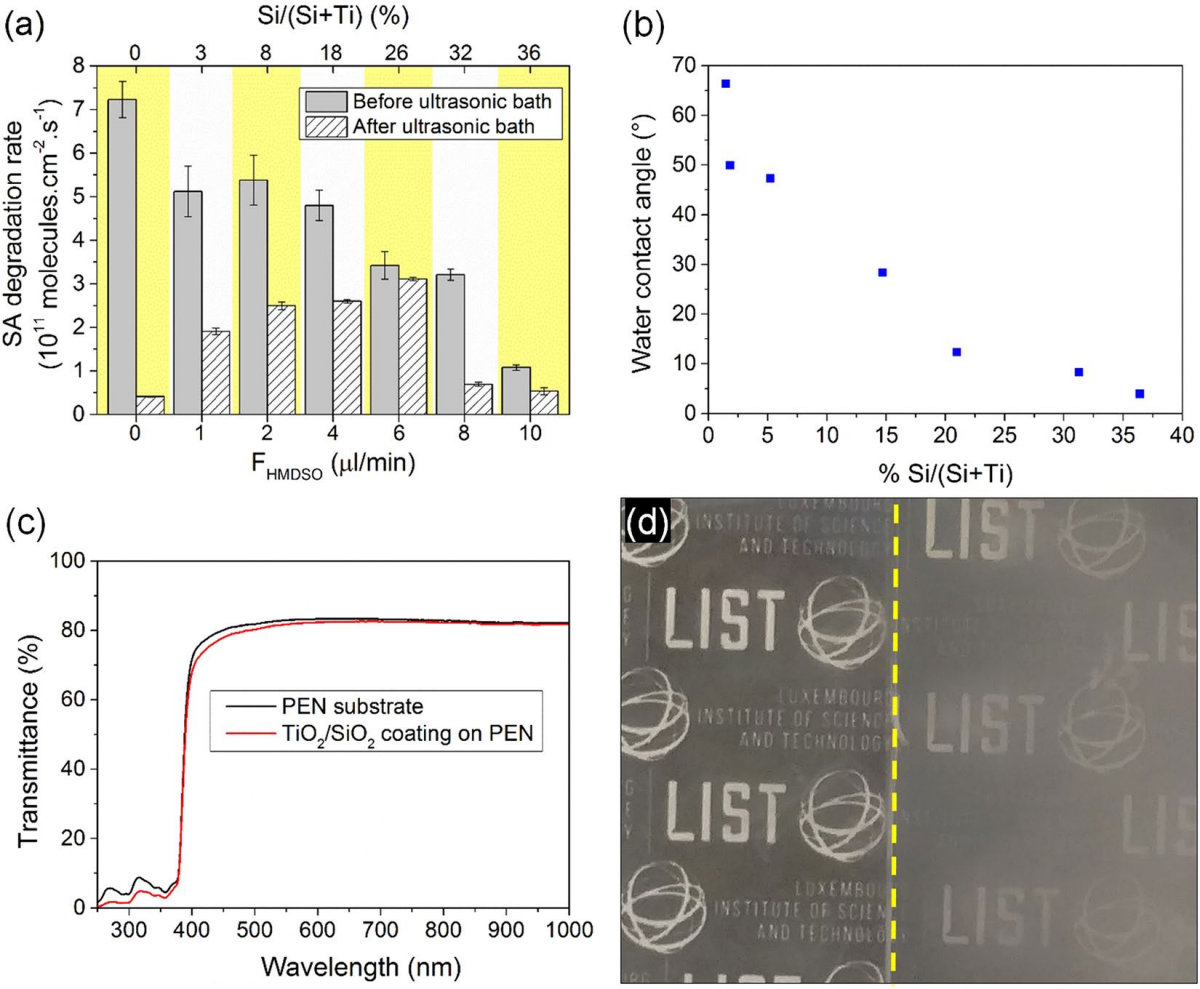
(**a**) Photocatalytic activity of anatase TiO_2_/SiO_2_ nanocomposite coatings for different Si/(Si + Ti) ratio before and after immersion in an ultrasonic bath for 7 hours. (**b**) Water contact angle of the as-deposited films as a function of the Si/(Si + Ti) ratio. (**c**) Transmission spectra in UV-Vis of an uncoated PEN foil and a PEN foil coated with anatase TiO_2_/SiO_2_ nanocomposite coating elaborated from a F_HMDSO_ = 10 µL·min^−1^ (**d**) Optical image of an uncoated PEN foil (right) and a PEN foil coated with anatase TiO_2_/SiO_2_ nanocomposite coating (left) elaborated from a F_HMDSO_ = 10 µL·min^−1^ exposed to a water steam.

The photocatalytic activity of the anatase TiO_2_/SiO_2_ nanocomposite coatings under UV light was assessed for each of the prepared coatings. Figure 5a shows the photodegradation of stearic acid (SA) over the deposited nanocomposite coatings expressed as degradation rate (degraded molecules per surface unit per time unit (molec.cm^−2^.s^−1^)). The highest stearic acid (SA) degradation rate, *i*.*e*. 7.2 × 10^11^ molec.cm^−2^.s^−1^, was measured for the pure TiO_2_ coating ([HMDSO] = 0 µL·min^−1^). Addition of HMDSO into the post-discharge induces a decrease of the photocatalytic activity of the anatase TiO_2_/SiO_2_ nanocomposite coatings. Unsurprisingly, the highest flow of HMDSO, i.e. [HMDSO] = 10 µL·min^−1^, leading to the lowest photocatalytic activity, i.e. 10^11^ molec.cm^−2^.s^−1^.

However, immersion of the coatings for 7 hours in an ultrasonic bath entirely reshuffled their photocatalytic activity performance ranking, highlighting strong discrepancies in the durability of the coatings (Fig. 5a). Interestingly, the SA degradation rate of anatase TiO_2_/SiO_2_ nanocomposite coating elaborated from a 6 µL·min^−1^ HMDSO delivery rate remained unchanged, *ca*. 3 × 10^11^ molec.cm^−2^.s^−1^. This contrasts with the photocatalytic activity of all the others samples, which was reduced in various proportions (Fig. 5a). Indeed, the photocatalytic activity of the anatase TiO_2_ coating and the anatase TiO_2_/SiO_2_ nanocomposite coatings elaborated from HMDSO flow rates lower than 4 µL·min^−1^ were reduced after ultrasonic bath from −95% to −50%, respectively (Fig. 5a). This results clearly highlights the benefit of the proposed nanocomposite approach, which allow to maintain a decent photocatalytic activity that remain unaltered even after sonication for 7 hours. Indeed, the anatase TiO_2_ particles, formed through homogeneous gas phase reactions induced by the high reactivity of the upper region of the plasma post-discharge, are yielding to loosely attached clusters on the surface. The simultaneous growth of a SiO_2_ layer allows the embedment of the anatase TiO_2_ particles in a mechanically resistant anatase TiO_2_/SiO_2_ nanocomposite coating with durable photocatalytic properties.

On the other hand, anatase TiO_2_/SiO_2_ nanocomposite coatings elaborated from HMDSO flow rates higher than 8 µL·min^−1^ also displayed a strong decrease of their photocatalytic activity, i.e. −50% to −75%, after 7 hours of immersion in an ultrasonic bath. This is not surprising as if the addition of a small amount of HMDSO promote the formation of an adherent matrix coating, excess of reactants in the gas phase favor the formation of particles. The gas phase reactions, exploited for the formation of the anatase TiO_2_ nanoparticles (F_TTIP_ = 6 µL·min^−1^)^10^ have to be prevented for the SiO_2_ precursor as they lead to the formation of more powdery coatings with lower stability and adhesion.

In addition to affording a better mechanical stability to the deposited films, the addition of the HMDSO precursor ensures a lowering of the water contact angle (WCA) of the as-deposited films (Fig. 5b). While the WCA of the pure TiO_2_ coating was evaluated at 66°, WCA as low as 5° was measured for the films with the highest content of silicon. This super-hydrophilic behavior is expected to benefit from the photocatalytic properties observed thanks to a better wetting of the surface of the TiO_2_/SiO_2_ nanocomposite coatings. However, the photocatalytic degradation ensured by the anatase TiO_2_ clusters is an asset to the durable super-hydrophilicity of the TiO_2_/SiO_2_ nanocomposite coatings.

It is also worth noting that the photocatalytic, super-hydrophilic and adherent TiO_2_/SiO_2_ nanocomposite coatings are also optically transparent. UV-visible transmission spectra (Fig. 5c) show a transmittance of the PEN with the coating very close to the uncoated PEN. As stated above, the combination of high transparency with durable photocatalytic and super-hydrophilic properties is particularly desirable for permanent anti-fogging coatings. In order to reach the potential of the approach developed for the anti-fogging application, both uncoated and coated polymer foils (i.e. PEN foils) were exposed to steam. As illustrated in Fig. 5d, a significant anti-fogging effect was achieved for the PEN foil coated with an anatase TiO_2_/SiO_2_ nanocomposite coating when compared to an uncoated PEN foil.

The contemporaneously but spatially separated injections of a TiO_2_ and an SiO_2_ precursor into the stream of an atmospheric pressure blown-arc discharge torch provide a convenient route towards the high-rate deposition of anatase TiO_2_/SiO_2_ nanocomposite coatings on both 2D and 3D substrates. In contrast with previously reported CVD approaches^12,13,22–24^, the anatase TiO_2_ phase is synthesized remotely from the substrate’s surface, making the developed method suitable for the coating of heat-sensitive substrates (Fig. 5b), including polymer lenses and organic electronic displays. The consecutive immobilization of the TiO_2_ nanoparticles into a silica matrix ensures the mechanical stability of the resulting anatase TiO_2_/SiO_2_ nanocomposite coatings. As a consequence, the photocatalytic activity of the anatase TiO_2_/SiO_2_ nanocomposite coatings, although lower than for the pure TiO_2_ coating (i.e. −50%), is still significant and more importantly, is fully retained even after prolonged sonication. In addition to the reported anti-fogging and self-cleaning potential of our transparent and multifunctional coatings, it may also find applications in anti-bacterial^25^ or effluent detoxification surfaces^26^.

## Conclusions

Multifunctional (anti-fogging, self-cleaning and high optical transparency) anatase TiO_2_/SiO_2_ nanocomposite coatings have been deposited on polymer substrates using an easily up-scalable and low-temperature atmospheric-pressure PECVD method. The atmospheric-pressure blown arc discharge torch, allowed the simultaneous synthesis of anatase TiO_2_ nanoparticles and their subsequent deposition in an amorphous SiO_2_ like matrix with a high deposition rate. Unsurprisingly, the photocatalytic properties on the anatase TiO_2_/SiO_2_ nanocomposite coatings, which were still significant, were found to be lower than those on the pure TiO_2_ coating. However, after extended sonication, the photocatalytic performances of the anatase TiO_2_/SiO_2_ nanocomposite coating (F_HMDSO_ = 6 µL·min^−1^) were fully retained. This is in contrast with the photocatalytic property drops observed for the other coating compositions after sonication. This applies in particular to the pure TiO_2_ coating that exhibited a severe drop in its photocatalytic properties. The hydrophilicity of the films was also enhanced with WCA as low as 5° for the as-deposited anatase TiO_2_/SiO_2_ nanocomposite coating prepared from the highest HMDSO delivery rate. As a consequence, this coating exhibited an efficient and durable anti-fogging behavior. All the anatase TiO_2_/SiO_2_ nanocomposite coatings were optically transparent and combined three functional properties (photocatalysis/self-cleaning, super-hydrophilicity/anti-fogging and optical transparency) within the same layer. This made our AP-PECVD approach (the blown-arc discharge torch equipment implemented on a 6-axis robot) a promising alternative to the robust transparent, anti-fogging and self-cleaning coatings for polymer-based lenses and displays, which are mass-produced at low temperatures and low atmospheric pressure.

The method developed for the simultaneous synthesis and low-temperature and high rate deposition of anatase TiO_2_–based thin films is certainly not specific to the thin film composition or applications described. It could be adapted well to the formation and consecutive immobilization of various crystalline metal oxides (e.g. ZnO, SnO_2_ or magnetic Fe_2_O_3_)^27^ or noble metal (e.g. Ag, Au, Pd and Pt) nanoparticles^28^. Similarly, the matrix material could easily be replaced by one of the numerous organic^29^ or inorganic coatings^30^ available via AP-PECVD. The wide range of coating combinations accessible, associated to the ability of the described plasma-based method to coat various substrate shapes (e.g. 3D) and materials (e.g. polymers), could allow the development of new applications. Interestingly, the *in-situ* synthesis and deposition of nanoparticles in the same process enables the handling of these potentially harmful materials to be avoided.

## Methods

### Atmospheric plasma deposition setup and materials

The plasma torch spot (ULS) from AcXys Technologies used in this work is described in detail in a previous paper^12^. In brief, a sinusoidal electrical signal with a frequency of 100 kHz and 600 W power is applied to the Hafnium electrode to generate a gliding arc discharge. The nitrogen gas flow through the plasma discharge, maintained at 30 L·min^−1^, allows the plasma discharge to be blown away. Titanium tetra-isopropoxide (TTIP, Sigma Aldrich, 98%) was used as a titanium-based precursor and hexamethyldisiloxane (HMDSO, Sigma Aldrich, 98%) as a silicon-based precursor. The respective flow of these precursors was controlled with a syringe pump. The TTIP flow was maintained constant at 6 µL·min^−1^ and the HMDSO flow was varied from 0 to 10 µL·min^−1^. Both precursors were injected through an ultrasonic nebulization nozzle (Sono-Tek Corporation) operating at a frequency of 120 kHz and 2 W power. The precursors’ mists, formed by each of the ultrasonic nozzles, with a droplet diameter of between 10 and 20 µm, were transported and introduced into the post-discharge zone at different positions using 3 L·min^−1^ (TTIP) and 5 L·min^−1^ (HMSDO) gas flows of nitrogen (Fig. 1a). To prevent any condensation on the walls, the TTIP injection line was heated to 90 °C. Poly(ethylene 2,6-naphthalate) (PEN) foils (100 µm thickness) and silicon wafers (100, double side polished, intrinsic, 270 µm thickness) were used as substrates. The plasma torch was maintained by a 6-axis robot (Fanuc LR mate) perpendicular to the substrate at a distance of z = 10 mm between the exit of the tube and the surface, and was moved along the X and Y axes at a speed of 30 mm.s^−1^ (Fig. 1b). The PEN and Si substrates were coated by successive parallel passes achieved by the robot movements, with increments of 1 mm along the Y-axis between each pass.

### Thin film characterizations and photocatalytic activity measurements

The Raman spectra were recorded with a Renishaw inVia micro-Raman spectrometer at an excitation wavelength of 532 nm with a laser power of approximately 0.44 mW focused on a 1 µm^2^ spot. The morphology and thickness of the coatings was observed by the Hitachi U70 Scanning Electron Microscope at a magnification of 50,000. To avoid any charging effect of the electron beam, the samples were first coated with a thin platinum ca. 5 nm film via magnetron sputtering deposition. XPS analyses were performed on a Kratos Axis Ultra DLD instrument using a monochromatic Al Kα X-ray source (hν = 1486.6 eV) 20 eV pass energy for high-resolution spectra. The core level spectra of Ti 2p and Si 2p were referenced to C1s at 284.5 eV. Argon-sputtered cleaning at 3 keV and 2 mA was used for approximately 500 s in a scanning mode in order to clean the surface and to obtain the most representative information on the elemental composition in the bulk of the coating. The WCA measurements were obtained using a Kruss DSA16 EasyDrop USB contact angle meter. The volume of the de-ionized water drop used for the measurements was 3 µL. The droplet was put in contact of the surface with a 10 µl syringe and the angle between the tangent of the droplet and the surface was measured after 2 seconds. The optical properties of the coating deposited on polymer foil (i.e. PEN) were measured in transmission mode using a Perkin Elmer Lambda 950 UV-Visible spectrometer equipped with a deuterium (UV) and tungsten (Vis) lamp and a photomultiplier. The photocatalytic activity measurements were done on coatings deposited on a double side polished undoped silicon wafer. 5 µl of steric acid diluted to 0.05 M in methanol was deposited on the coated wafer using a spin coater rotating at 1,000 rpm for 30 s. Afterward, the sample was placed in a box 20 cm away from an 8 W UV light with a wavelength of 254 nm. The kinetic degradation of the stearic acid (SA) was followed by regular analysis using Fourier transform infrared spectroscopy (FTIR) in transmission mode (Bruker Vertex 70) thanks to the decrease of the CH_2_-CH_3_ absorption band in the 2,800-3,000 cm^−1^ range during its illumination duration. The SA degradation rate (R_SA_) was estimated from the linear regression of the corresponding FTIR integrated areas vs. irradiation time degradation curves (slop of the linear regression) expressed in molecules of SA cm^−2^ using the conversion factor of 9.7 × 10^15^ molecules SA cm-2 ≡ 1 cm^−1^^31^. The mechanical stability and photocatalytic activity durability of the deposited coatings were assessed by submitting coated samples to an ultrasonic bath for 7 hours, where they were immersed in ethanol, before carrying out a new photocatalytic activity measurement. The anti-fogging property of the coating was investigated and compared to uncoated substrates by submitting the surfaces to steam created by a water tank at 65 °C placed 5 cm below the samples.

## Electronic supplementary material

Supplementary information

## References

[1] Tompson CS, Fleming RA, Zou M (2013). Transparent self-cleaning and antifogging silica nanoparticle films. Sol. Energy Mater. Sol. Cells.

[2] Shokuhfar A, Alzamani M, Eghdam E, Karimi M, Mastali S (2012). SiO2-TiO2 Nanostructure Films on Windshields Prepared by Sol-Gel Dip-Coating Technique for Self-Cleaning and Photocatalytic Applications. Nanosci. Nanotechnol..

[3] Chevallier P, Turgeon S, Sarra-Bournet C, Turcotte R, Laroche G (2011). Characterization of multilayer anti-fog coatings. ACS Appl. Mater. Interfaces.

[4] Chen Y (2012). Transparent superhydrophobic/superhydrophilic coatings for self-cleaning and anti-fogging. Appl. Phys. Lett..

[5] Tricoli A, Righettoni M, Pratsinis SE (2009). Anti-fogging nanofibrous sio2 and nanostructured sio 2-tio2 films made by rapid flame deposition and *in situ* annealing. Langmuir.

[6] Park KC (2012). Nanotextured silica surfaces with robust superhydrophobicity and omnidirectional broadband supertransmissivity. ACS Nano.

[7] Liu F, Shen J, Zhou W, Zhang S, Wan L (2017). *In situ* growth of TiO_2_/SiO_2_ nanospheres on glass substrates via solution impregnation for antifogging. RSC Adv..

[8] Li X, He J (2013). Synthesis of Raspberry-Like SiO2−TiO2 Nanoparticles toward Antire fl ective and Self-CleaningCoatings. ACS Appl. Mater. Interface.

[9] Saxena N, Naik T, Paria S (2017). Organization of SiO_2_ and TiO_2_ Nanoparticles into Fractal Patterns on Glass Surface for the Generation of Superhydrophilicity. J. Phys. Chem. C.

[10] Dembele A (2011). Deposition of Hybrid Organic-Inorganic Composite Coatings using an Atmospheric Plasma Jet System. J. Nanosci. Nanotechnol..

[11] Boscher ND, Choquet P, Duday D, Verdier S (2010). Chemical compositions of organosilicon thin films deposited on aluminium foil by atmospheric pressure dielectric barrier discharge and their electrochemical behaviour. Surf. Coatings Technol..

[12] Maurau R (2013). Atmospheric pressure, low temperature deposition of photocatalytic TiOx thin films with a blown arc discharge. Surf. Coatings Technol..

[13] Gazal Y (2016). Multi-structural TiO2 film synthesised by an atmospheric pressure plasma-enhanced chemical vapour deposition microwave torch. Thin Solid Films.

[14] Quesada-González M, Boscher ND, Carmalt CJ, Parkin IP (2016). Interstitial Boron-Doped TiO2 Thin Films: The Significant Effect of Boron on TiO2 Coatings Grown by Atmospheric PressureChemical Vapor Deposition. ACS Appl. Mater. Interfaces.

[15] Baba K, Bulou S, Choquet P, Boscher ND (2017). Photocatalytic Anatase TiO2 Thin Films on Polymer Optical Fiber Using Atmospheric-PressurePlasma. ACS Appl. Mater. Interface.

[16] Quesada-González M (2017). Interstitial boron-doped anatase TiO_2_ thin-films on optical fibres: atmospheric pressure-plasma enhanced chemical vapour deposition as the key for functional oxide coatings on temperature-sensitive substrates. J. Mater. Chem. A.

[17] Boscher ND, Choquet P, Duday D, Verdier S (2011). Influence of cyclic organosilicon precursors on the corrosion of aluminium coated sheet by atmospheric pressure dielectric barrier discharge. Surf. Coatings Technol..

[18] Zhang WL, Zhang S, Yang M, Chen TP (2010). Microstructure of magnetron sputtered amorphous SiOx films: Formation of amorphous Si core-shell nanoclusters. J. Phys. Chem. C.

[19] Baba K (2017). Significance of a Noble Metal Nanolayer on the UV and Visible Light Photocatalytic Activity of Anatase TiO2 Thin Films Grown from a Scalable PECVD/PVD Approach. ACS Appl. Mater. Interfaces.

[20] Paoluzi, A. & Turilli, G. XPS and XAES Study of TiO_2_-SiO_2_ Mixed Oxide System. **92**, 3–6 (1990).

[21] Brassard D, Sarkar DK, El Khakani MA, Quellet L (2004). Tuning the electrical resistivity of pulsed laser deposited TiSiO x thin films from highly insulating to conductive behaviors. Appl. Phys. Lett..

[22] Boscher ND (2014). Photocatalytic anatase titanium dioxide thin films deposition by an atmospheric pressure blown arc discharge. Appl. Surf. Sci..

[23] Sotelo-Vazquez C, Quesada-Cabrera R, Darr JA, Parkin IP (2014). Single-step synthesis of doped TiO2 stratified thin-films by atmospheric-pressure chemical vapour deposition. J. Mater. Chem. A.

[24] Li D (2016). Structural and Optical Properties of PECVD TiO2–SiO2 Mixed Oxide Films for Optical Applications. Plasma Process. Polym..

[25] Bonetta S, Bonetta S, Motta F, Strini A, Carraro E (2013). Photocatalytic bacterial inactivation by TiO2-coated surfaces. AMB Express.

[26] Henderson MA (2011). A surface science perspective on TiO2 photocatalysis. Surf. Sci. Rep..

[27] Bouclé J, Ravirajan P, Nelson J (2007). Hybrid polymer–metal oxide thin films for photovoltaic applications. J. Mater. Chem..

[28] Koo IG, Lee MS, Shim JH, Ahn JH, Lee WM (2005). Platinum nanoparticles prepared by a plasma-chemical reduction method. J. Mater. Chem..

[29] Hilt F (2014). Atmospheric pressure plasma-initiated chemical vapor deposition (AP-PiCVD) of poly(diethylallylphosphate) coating: A char-forming protective coating for cellulosic textile. ACS Appl. Mater. Interfaces.

[30] Baba K, Lazzaroni C, Nikravech M (2014). Growth of ZnO thin films by spray plasma technique: Correlation between spectroscopic measurements and film properties. Plasma Chem. Plasma Process..

[31] Mills A, Wang J (2006). Simultaneous monitoring of the destruction of stearic acid and generation of carbon dioxide by self-cleaning semiconductor photocatalytic films. J. Photochem. Photobiol. A Chem..

