# Supplementary material for: Transparent anti-fogging and self-cleaning TiO2/SiO2 thin films on polymer substrates using atmospheric plasma
Source: Sci Rep. 2018 Jun 25;8:9603. doi: 10.1038/s41598-018-27526-7 (PMC6018401; doi:10.1038/s41598-018-27526-7)
Supplement: Supplementary file 1 — Supplementary information [file 41598_2018_27526_MOESM1_ESM.docx]

**Transparent anti-fogging and self-cleaning TiO_2_/SiO_2_ thin films on polymer substrates using atmospheric plasma**

***Jean-Baptiste Chemin^1*^, Simon Bulou^1^, Kamal Baba^1^, Charly Fontaine^1^, Thierry Sindzingre^2^, Nicolas D. Boscher^1^ and Patrick Choquet^1^***

^1^Materials Research and Technology Department, Luxembourg Institute of Science and Technology, 5 Avenue des Hauts-Fourneaux, L-4362 Esch-sur-Alzette, Luxembourg.

^2^AcXys Technologies, 50 bis Rue des Vingt Toises, 38950 Saint Martin le Vinoux, France

* Correspondence to [jean-baptiste.chemin@list.lu](mailto:jean-baptiste.chemin@list.lu)

# **Supplementary information**


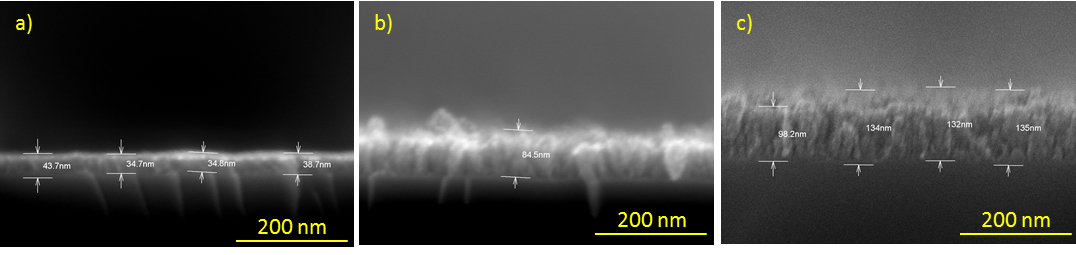


Figure S1 : SEM on cross section obtain at a magnification of 200 000 for coating made with 6µl.min^-1^of TTIP, without HMDSO (a) with 2µl.min^-1^ of HMDSO (b) and 6µl.min^-1^of HMDSO (c)
